# A social robot connected with chatGPT to improve cognitive functioning in ASD subjects

**DOI:** 10.3389/fpsyg.2023.1232177

**Published:** 2023-10-05

**Authors:** Francesca Bertacchini, Francesco Demarco, Carmelo Scuro, Pietro Pantano, Eleonora Bilotta

**Affiliations:** ^1^Department of Mechanical, Energy and Management Engineering, University of Calabria, Rende, Italy; ^2^Laboratory of Cognitive Psychology and Mathematical Modelling, University of Calabria, Rende, Italy; ^3^Department of Physics, University of Calabria, Rende, Italy

**Keywords:** social robotics, neurodevelopmental disorders, Autism Spectrum Disorders, facial emotions recognition, educational scenarios, physical disabilities

## Abstract

Neurodevelopmental Disorders (NDDs) represent a significant healthcare and economic burden for families and society. Technology, including AI and digital technologies, offers potential solutions for the assessment, monitoring, and treatment of NDDs. However, further research is needed to determine the effectiveness, feasibility, and acceptability of these technologies in NDDs, and to address the challenges associated with their implementation. In this work, we present the application of social robotics using a Pepper robot connected to the OpenAI system (Chat-GPT) for real-time dialogue initiation with the robot. After describing the general architecture of the system, we present two possible simulated interaction scenarios of a subject with Autism Spectrum Disorder in two different situations. Limitations and future implementations are also provided to provide an overview of the potential developments of interconnected systems that could greatly contribute to technological advancements for Neurodevelopmental Disorders (NDD).

## 1. Introduction

Neurodevelopmental Disorders (NDDs) pose significant challenges to individuals, families, and society, both in terms of healthcare and economic burden. Advances in technology, including Artificial Intelligence (AI) and digital technologies, hold promise for addressing the assessment, monitoring, and treatment needs of individuals with NDDs. However, further research is required to determine the effectiveness, feasibility, and acceptability of these technologies within the context of NDDs, and to overcome the associated implementation challenges. In this paper, we present an innovative application of social robotics, utilizing a Pepper robot connected to the OpenAI system (Chat-GPT), to facilitate real-time dialogue and image generation as opposed to the traditional use of dynamic generative systems (Bilotta et al., 2009, 2010, 2021; Adamo et al., 2010; Bertacchini et al., 2010, 2012, 2013, 2022a,b,c, 2023; Gabriele et al., 2017). By combining these technologies, we aim to enhance the interaction experience and promote communication in individuals with Autism Spectrum Disorder (ASD), particularly those with low-medium functioning capabilities. We begin by describing the general architecture of the interconnected system, outlining the components and their functionalities. The Pepper robot serves as a social companion, employing speech recognition and synthesis, facial expression capabilities, and body language to create a responsive and engaging interaction environment. The integration with the OpenAI system enables real-time dialogue initiation, utilizing natural language understanding to interpret and generate meaningful responses. Furthermore, we present two simulated interaction scenarios, showcasing the potential applications of the system in different contexts. These scenarios involve the engagement of an individual with low functioning ASD, addressing communication challenges and problem-solving abilities. The scenarios aim to foster social interaction, imaginative play, and cognitive skills development, emphasizing the personalized and adaptable nature of the robotic interactions in connection with AI superpower (Wolfram, 2020). The presented scenarios are toy-examples, and the effectiveness of the system may vary across individuals with NDDs due to the heterogeneity of the disorders. Additionally, further research is required to validate and optimize the system's performance in real-world settings, considering factors such as individual differences, environmental considerations, and user preferences. The paper examines the current state of evidence regarding the effectiveness, feasibility, and acceptability of AI and digital technologies in the assessment, monitoring, and treatment of NDD. We will summarize the available literature on the use of AI and digital technologies in NDD, focusing on their clinical and economic impact, and their potential to improve the quality and accessibility of care. Finally, we will discuss the challenges and opportunities associated with the use of AI and digital technologies in NDD and provide recommendations for future research in this area. Furthermore, this work highlights the potential of interconnected systems, combining social robotics, AI, and image generation technologies, to address the needs of individuals with NDDs, specifically with Autism Spectrum Disorder (ASD) subjects. It underlines the importance of further research to assess the effectiveness, feasibility, and acceptability of these technologies, as well as the need for ongoing developments and adaptations to offer to the diverse needs of individuals with ASD. The focus of this paper is to present an innovative educational approach aimed at addressing the unique needs of individuals with Autism Spectrum Disorder (ASD). Specifically, we propose a comprehensive set of lessons designed to harness the capabilities of ChatGPT, an advanced conversational AI, and integrate them with the use of images to enhance the development of emotion recognition skills in individuals with ASD and the development of cognitive skills such as problem solving. We conceptualize this integration as a path of educational robotics, blending cutting-edge technology with educational methodologies to create an engaging and effective learning experience. By combining the power of artificial intelligence, visual stimuli, and interactive robotics, we aim to provide a novel and promising avenue for supporting individuals with ASD in their neurodevelopmental journey. The paper is organized as follows. In Section 2, we provide a synthesis of NDDs and technological trends and research in this field. Section 3 provides the examination of a variety of tools and techniques designed to assist individuals with neurological disorders in various aspects of their lives. Sections 4 is about social robotics and the use of these technologies in the context of ASD. Section 5 deals about practical steps on how to connect OpenAI with the robot Pepper, delineating a new way of interacting with the subject.

## 2. Neurodevelopmental disorders and AI related technologies

Neurodevelopmental disorders (NDDs) are a group of conditions that have their origins in childhood and affect an individual's ability to function in personal, social, academic, or work settings. These conditions include Attention Deficit Hyperactivity Disorder (ADHD), Autism Spectrum Disorder (ASD), and specific learning disorders, among others. The chronic nature of these conditions represents a significant healthcare and cost burden for families and society. Patients with NDD often experience delays in receiving a diagnosis and treatment, which can negatively impact their social, interpersonal, and academic performance.

Technology offers the potential to provide more efficient and accessible solutions for the assessment, monitoring, and treatment of NDD. Artificial Intelligence (AI) and digital technologies such as telemedicine, virtual reality, computerized cognitive training, and wearable devices are examples of technologies that have shown promise in improving the accessibility and quality of care for individuals with NDD.The purpose of this paper is to review the current state of evidence regarding the effectiveness, feasibility, and acceptability of AI and digital technologies in the assessment, monitoring, and treatment of NDD. We will summarize the available literature on the use of AI and digital technologies in NDD, focusing on their clinical and economic impact, and their potential to improve the quality and accessibility of care. Finally, we will discuss the challenges and opportunities associated with the use of AI and digital technologies in NDD and provide recommendations for future research in this area.

**Neurodevelopmental disorders (NDD)** are characterized by deficits in development that impair personal, social, academic, or work functioning. These conditions often have their origins in childhood and persist into adulthood, representing a significant healthcare and cost burden for families and society (American Psychiatric Association, 2013; Buescher et al., 2014; Bertacchini et al., 2021). The most common NDDs include ADHD, ASD, and specific learning disorders (Simmons et al., 2009). NDD conditions encompass a diverse range of neurodevelopmental disorders and disabilities that can significantly impact an individual's learning, social interaction, and overall functioning. Some common NDD conditions include:

**Autism Spectrum Disorder**: ASD is a complex neurodevelopmental disorder characterized by difficulties in social communication and interaction, restricted interests, and repetitive behaviors. Individuals with ASD may have varying levels of cognitive abilities and require individualized support to navigate academic and social environments effectively.
**Attention deficit hyperactivity disorder**: ADHD is a neurodevelopmental disorder characterized by difficulties with attention, impulsivity, and hyperactivity. Students with ADHD often struggle with maintaining focus, organizing tasks, and following instructions, which can affect their academic performance and social interactions.
**Specific learning disabilities**: SLD refers to difficulties in specific areas of learning, such as reading (dyslexia), writing (dysgraphia), or mathematics (dyscalculia). These conditions can significantly impact an individual's ability to acquire and apply specific academic skills despite having average or above-average intelligence in other areas.
**Intellectual disability**: ID is a condition characterized by significant limitations in intellectual functioning and adaptive behavior. Individuals with ID may experience challenges in areas such as communication, self-care, and social skills, requiring specialized support and accommodations.
**Speech and language disorders**: These disorders involve difficulties in speech production, language comprehension, or communication. Conditions such as expressive language disorder, phonological disorder, or stuttering can affect an individual's ability to express themselves verbally and understand others effectively.
**Physical disabilities**: Physical disabilities, such as cerebral palsy, muscular dystrophy, or spina bifida, can impact mobility, fine motor skills, and coordination. These conditions may require assistive technologies and adaptations in the educational environment to ensure accessibility and promote active participation. It's crucial to recognize that each NDD condition is unique, and individuals within each category can exhibit a wide range of abilities and challenges. Understanding the specific characteristics and needs associated with different NDD conditions is vital for developing effective inclusive educational practices and providing appropriate support to students.

One of the major challenges associated with NDD is delayed diagnosis and treatment, which can have negative effects on an individual's social, interpersonal, and academic performance. For example, a recent randomized controlled trial found that 40% of families referred for an ADHD evaluation were still awaiting diagnosis six months after their initial assessment (Hollis et al., 2018). Similarly, individuals with Tourette's syndrome often face limited access to behavioral therapy for tic management, with many receiving less than the recommended number of therapy sessions (Cuenca et al., 2015). The chronic nature of NDDs also results in a significant economic burden for families and society. For instance, the estimated annual cost of autism in the United States is expected to reach $461 billion by 2025, including medical care, support services, housing, transportation, education, and lost productivity (Hurley-Hanson et al., 2020). Similarly, the estimated annual cost of ADHD in the United States ranges from $143 to 266 billion, with the majority of the burden represented by lost income and productivity for adults, as well as healthcare and education expenses (Doshi et al., 2012). Given the high prevalence and economic burden of NDDs, there is a growing need for more effective and efficient healthcare services. Technology, including AI and digital technologies, offers promising solutions for the assessment, monitoring, and treatment of NDDs (Bilotta et al., 2007; Torous and Roberts, 2017; Valentine et al., 2020). For instance, virtual reality, computerized cognitive training, and wearable devices have shown potential to improve accessibility and quality of care for individuals with NDDs (Bilotta et al., 2003; Hollis et al., 2017; Havdahl et al., 2021).

Artificial Intelligence (AI) and related technologies have the potential to provide valuable assistance to individuals with NDD conditions. These technologies can offer personalized support, enhance communication abilities, facilitate learning, and promote greater independence. Here are some ways AI and related technologies can help:

1. Personalized learning: AI-powered educational tools can adapt to an individual's learning style, pace, and abilities. They can provide customized learning materials, adaptive assessments, and interactive activities tailored to the specific needs of each student.
2. Communication support: AI-based communication aids, such as symbol-based systems or text-to-speech applications, can facilitate communication for individuals with speech or language disorders. These tools enable them to express their thoughts, needs, and ideas effectively.
3. Assistive technologies: AI can enhance accessibility by enabling individuals with physical disabilities to control devices or interact with their environment through voice commands, gesture recognition, or eye-tracking technologies. This promotes greater independence and participation in daily activities.
4. Behavioral and emotional support: AI algorithms can analyze patterns of behavior and provide real-time feedback or interventions to help individuals manage challenging behaviors or regulate their emotions. These technologies can assist individuals with conditions like ADHD or autism in improving self-regulation skills.
5. Social skills training: AI-based virtual reality simulations can create safe and controlled environments for individuals to practice social interactions and develop social skills. These simulations offer opportunities for learning and self-confidence building in contexts that might otherwise be challenging or overwhelming.
6. Data analysis and insights: AI algorithms can process large amounts of data, such as educational records or behavioral assessments, to identify patterns, trends, and personalized recommendations for intervention. This can assist educators and therapists in making informed decisions and tailoring interventions to each individual's needs.

A summary for Specific NDD Conditions and AI and related technologies is reported in Table 1.

**Table 1:** Specific NDD conditions and AI and related technologies.

**NDD condition**	**AI and related technologies**
Autism spectrum disorder	AI-based communication aids, social skills training in virtual reality, personalized learning platforms tailored to specific needs, data analysis for behavior monitoring and intervention planning, Human robot interaction, social robotics, social robotics and AI large Language Model
Attention deficit hyperactivity disorder	AI-based adaptive learning tools, text-to-speech and speech recognition technologies, personalized instruction for targeted skill development
Specific learning disabilities (e.g., dyslexia, dysgraphia, dyscalculia)	AI-based adaptive learning tools, text-to-speech and speech recognition technologies, personalized instruction for targeted skill development
Intellectual disability	AI-powered assistive technologies for communication and independent living, adaptive learning platforms, virtual reality simulations for life skills training
Speech and language disorders	AI-enabled assistive technologies for mobility and accessibility, smart home devices for environmental control, adaptive tools for computer access, personalized rehabilitation programs

This table provides a general overview of how AI and related technologies can assist individuals with specific NDD conditions. The actual applications and tools available may vary, and it's essential to consider individual needs and consult with professionals to determine the most suitable technologies for each person. However, the effectiveness, feasibility, and acceptability of these technologies in the assessment, monitoring, and treatment of NDDs remain uncertain. While some systematic reviews have found promising evidence for the use of digital technologies in NDDs, the quality and size of available studies are often limited (Hall et al., 2016; Hollis et al., 2017). Moreover, there are challenges associated with the implementation of these technologies, including limited access to qualified therapists and inadequate clinical time to provide optimal care (Hall et al., 2016).

### 2.1. Literature review on AI tools for NDD

Barua et al. (2022) discusses the high prevalence of neurodevelopmental disorders (NDDs) and mental health disorders (MHDs) in children and the difficulties they face in learning due to social and communication deficits. The authors argue that timely and effective interventions are necessary to improve outcomes for these children. The review focuses on the use of AI-assisted tools, which have been developed using machine learning models, to address the learning challenges faced by students with NDDs. The authors provide evidence that AI tools can successfully improve social interaction and supportive education. However, they also highlight the limitations of existing AI tools and provide recommendations for the development of future AI tools that focus on providing personalized learning for individuals with NDDs. The review provides valuable insights into the use of AI in enhancing education for children with NDDs and highlights the need for further research in this area. Uddin et al. (2019) discuss the potential of using artificial intelligence (AI) in precision medicine for neurodevelopmental disorders (NDDs). Precision medicine aims to personalize treatment and intervention for each patient based on their unique biological and environmental factors. While AI algorithms have been successful in predicting risk for certain diseases, such as cancer and cardiovascular disease, progress has been slower in NDDs, which include conditions like Autism Spectrum Disorder (ASD) and intellectual disability (ID). This is due to the phenotypic and etiologic heterogeneity of NDDs, as well as the variability of risk variants identified so far. The article explores the challenges in using AI for NDDs and potential strategies to overcome them, such as integrating genomic data with other biomarkers and developing more sensitive diagnostic tools.

Mengi and Malhotra (2021) explore the use of artificial intelligence (AI) techniques for the detection of socio-behavioral disorders (SBD), which is a subtype of neurodevelopmental disorders (NDDs). SBDs include conditions such as Autism Spectrum Disorder (ASD) and attention deficit hyperactivity disorder (ADHD). The traditional diagnosis of SBDs is based on qualitative parameters, which can be time-consuming. Automated diagnostic systems based on quantitative parameters can provide reliability, accuracy, and affordability. The article provides a thorough investigation of efforts on the subject of automated diagnostic systems using AI techniques based on biomarkers and multimodal data for early detection of ASD or ADHD. The studies of various automated AI-based diagnostic systems for the detection of ASD, ADHD, and comorbid ASD and ADHD are also presented in this research work. Finally, the article highlights the open issues existing in the literature that need to be explored further for providing more effective SBD analysis and presents an outline of proposed work which will aid in the diagnostic field of ASD, ADHD, and comorbid ASD and ADHD.

The paper of Vashisht and Jatain (2023) proposes a computer-aided approach for diagnosing neurodevelopmental disorders using mathematical and deep learning models. The framework is designed to identify four common neurodevelopmental disorders that often occur in the early phases of a child's life. The proposed system aims to provide suitable remedies and strategies to parents and teachers to help the child recover from the illness. According to these authors, neurodevelopmental disorders (NDDs) are a group of conditions that affect the development of the brain and nervous system, leading to a range of impairments in motor, cognitive, and social functioning. Diagnosing NDDs is a challenging task and often requires a comprehensive evaluation of the child's developmental history, behavioral symptoms, and clinical assessments. In this paper, we propose a novel approach for diagnosing NDDs using artificial intelligence (AI) techniques. Our proposed framework utilizes mathematical and deep learning models to diagnose four common NDDs, including Attention Deficit Hyperactivity Disorder (ADHD), Autism Spectrum Disorder (ASD), Language Disorders (LD), and Intellectual Disabilities (ID). The proposed system analyses various features, such as electroencephalogram (EEG), magnetic resonance imaging (MRI), and behavioral data, to identify the presence of NDDs. The system generates a report that includes the diagnosis, severity of the disorder, and recommended remedies and strategies for parents and teachers to help the child recover from the illness. The proposed system has the potential to revolutionize the diagnosis and treatment of NDDs by providing an accurate, objective, and efficient diagnostic tool for clinicians and caregivers.

The application of machine learning (ML) in the diagnosis and treatment of neurodevelopmental disorders (NDDs) in children is reported in Song et al. (2022). The authors highlight the increasing prevalence of NDDs among children and the challenges associated with early diagnosis and intervention. They discuss how ML technology, which combines artificial intelligence and medicine, has been used to perform early detection and prediction of diseases based on data mining. The paper reviews the progress made in the application of supervised and unsupervised learning tools in the diagnosis and treatment of NDDs in children. The authors suggest that ML offers new perspectives on the early diagnosis and treatment of NDDs, and could potentially reduce the burden on families and healthcare systems.

The study of Chen et al. (2020) aimed to investigate the possibility of identifying neuroimaging biomarkers that can distinguish patients with Autism Spectrum Disorder (ASD) from healthy controls using structural magnetic resonance imaging (sMRI) data. A novel 2-level histogram-based morphometry (HBM) classification framework was developed and applied to fourdatasets from the Autism Brain Imaging Data Exchange. The framework achieved an area under the curve (AUC) of >0.75 in each dataset, with the highest AUC of 0.849. The study identified ASD-related brain regions based on the sMRI images, including regions previously implicated in ASD, such as the frontal gyrus, temporal gyrus, cingulate gyrus, postcentral gyrus, precuneus, caudate, and hippocampus, as well as less well-known regions that may play unrecognized roles in ASD. The study suggests that it is possible to identify neuroimaging biomarkers for ASD using the more cost-effective sMRI images of the brain, and demonstrates the potential of applying data-driven artificial intelligence technology in the clinical setting of neurological and psychiatric disorders.

Alcañiz et al. (2019) focus on the use of virtual reality (VR) as a tool for the evaluation and diagnosis of Autism Spectrum Disorder (ASD). Traditionally, diagnostic tools for ASD have relied on qualitative criteria and observational information in low ecological validity contexts. However, there is a growing interest in using implicit measures based on unconscious processes to assess and diagnose ASD. These measures involve the acquisition and analysis of brain, physiological, and behavioral responses to stimuli, aiming to capture the behavioral structure of individuals with ASD. The complex relationship between physiological responses and the behavioral structure of individuals with ASD requires advanced signal processing techniques based on cognitive computation. Artificial intelligence (AI) techniques, such as machine learning and neurocomputing, have shown robustness in classifying complex cognitive constructs. VR offers the opportunity to recreate real-life situations with high sensory fidelity while individually controlling stimuli that influence human behavior. It also enables real-time measurement of human reactions to these stimuli. This review discusses the latest scientific and technological advancements in VR and its applications in the diagnosis of ASD. The authors conclude that VR is a valuable tool for ASD research, particularly in evaluating and diagnosing complex skills and competencies. The immersive and controlled nature of VR allows for more ecologically valid assessments, providing insights into the cognitive and behavioral characteristics of individuals with ASD.

The paper of Garzotto et al. (2018) explores the potential of integrating Wearable Virtual Reality (WVR) and bio-sensors for the treatment of individuals with Neurodevelopmental Disorders (NDD). The authors propose using wearable bio-sensors in conjunction with WVR applications to improve attention skills and autonomy in individuals with NDD. The information collected from the bio-sensors, along with interaction logs, can be visualized to help therapists monitor the patient's state and attention levels during a WVR experience. By comparing results from different sessions, therapists can assess the progress and improvements made by the patients. This approach complements traditional observation-based evaluation methods and clinical tests, and it supports evidence-based research on the effectiveness of Wearable VR for individuals with NDD. By integrating wearable bio-sensors with WVR, this approach has the potential to enhance therapy and provide valuable insights for the treatment of individuals with NDD.

In the study of Chen et al. (2020) the researchers aimed to develop a practical artificial intelligence (AI) tool for diagnosing and evaluating Autism Spectrum Disorder (ASD). They focused on identifying structural patterns in the brain that could serve as potential biomarkers for the diagnosis and evaluation of ASD in clinical settings. The researchers developed a novel classification framework based on a 2-level histogram-based morphometry (HBM) approach and used a 3D version of the histogram of oriented gradients (HOG) algorithm to extract features from structural magnetic resonance imaging (sMRI) data.

The study utilized four datasets from the Autism Brain Imaging Data Exchange to distinguish patients with ASD from healthy controls. The proposed HBM framework achieved high accuracy, with an area under the curve (AUC) exceeding 0.75 in each dataset. The highest AUC of 0.849 was observed in the ETH site. Comparisons with other algorithms showed improvements in accuracy, highlighting the effectiveness of the 3D HOG algorithm for ASD diagnosis. Additionally, the researchers identified ASD-related brain regions based on the sMRI images, including well-known regions implicated in ASD, such as the frontal gyrus, temporal gyrus, cingulate gyrus, postcentral gyrus, precuneus, caudate, and hippocampus. They also identified less explored regions that may play unrecognized roles in ASD, warranting further investigation. The findings of this study suggest the possibility of using neuroimaging biomarkers derived from cost-effective sMRI images to distinguish patients with ASD from healthy individuals. The research demonstrates the potential of AI technology in the clinical setting of neurological and psychiatric disorders, where subtle anatomical changes in the brain may not be readily visible to the human eye.

## 3. Tools and techniques for NDDs

Neurodevelopmental Disorder Technologies encompass a variety of tools and techniques designed to assist individuals with neurological disorders in various aspects of their lives. Here are some specific examples of these tools:

1. Communication aid devices: These devices include specialized software applications running on tablets or dedicated communication devices. They are designed to support individuals with communication difficulties, such as those with autism who may have challenges with verbal or non-verbal communication. These tools often offer visual aids, symbol-based communication systems, or text-to-speech capabilities to facilitate effective communication.
2. Augmentative and alternative communication systems: AAC systems encompass a wide range of tools and techniques that support or replace traditional spoken language for individuals with communication impairments. These systems can include communication boards, picture exchange communication systems (PECS), speech-generating devices, and mobile applications specifically designed for AAC purposes.
3. Behavior tracking and monitoring systems: These technologies utilize sensors and wearable devices to track and monitor an individual's behavior and activities. They can provide valuable insights into patterns of behavior, identify triggers for challenging behaviors, and help caregivers and therapists develop targeted intervention strategies. For instance, wearable devices may track physiological indicators like heart rate, sleep patterns, or emotional states to detect early signs of distress or agitation.
4. Virtual reality and augmented reality applications: VR and AR technologies have shown promise in providing immersive and interactive experiences for individuals with neurodevelopmental disorders. They can be used in therapeutic settings to simulate real-life situations and environments, helping individuals develop social skills, practice coping strategies, or desensitize themselves to sensory stimuli in a controlled manner.
5. Assistive technologies for daily living: These tools aim to support individuals with neurodevelopmental disorders in their daily activities and promote independence. Examples include smart home devices, reminder systems, task schedulers, and mobile applications that assist with time management, organization, and daily routines.
6. Sensory integration tools: Individuals with neurodevelopmental disorders often experience challenges with sensory processing. Sensory integration tools, such as sensory toys, weighted blankets, or noise-canceling headphones, can help individuals regulate their sensory experiences and promote a calmer and more focused state.

## 4. Social robotics

Social robotics is a field that explores the interaction between humans and robots with a focus on social and emotional elements. When it comes to ASD, social robotics has shown promise in providing innovative tools and interventions to support individuals with ASD in various aspects of their development and social interactions. Here are some key points regarding the use of social robotics in the context of ASD:

1. Social engagement: Social robots can engage individuals with ASD in social interactions by providing a predictable, structured, and non-threatening environment. These robots are designed to exhibit human-like behaviors, such as maintaining eye contact, displaying facial expressions, and engaging in turn-taking conversations. By interacting with social robots, individuals with ASD can develop and practice social skills in a controlled and supportive setting.
2. Communication support: Social robots can act as communication aids for individuals with ASD who have challenges with verbal or non-verbal communication. They can use visual displays, symbols, or speech output to facilitate communication, allowing individuals to express themselves and interact with others more effectively. The robots can also provide prompts, visual schedules, or social stories to help individuals navigate social situations.
3. Emotional regulation: Many individuals with ASD struggle with emotional regulation and understanding others' emotions. Social robots can be programmed to recognize and respond to emotional cues, providing support in managing emotions. They can teach emotional recognition, regulation techniques, and social problem-solving skills through interactive activities and games.
4. Personalized learning: Social robots can adapt their interactions and interventions based on individual needs and preferences. Through machine learning algorithms, robots can analyze an individual's responses and adjust their prompts or activities to provide personalized learning experiences. This adaptability can enhance engagement and promote skill development tailored to the specific challenges and strengths of each individual with ASD.
5. Promoting social initiations: Social robots can encourage individuals with ASD to initiate social interactions by presenting socially appropriate prompts, suggestions, or activities. By providing a supportive and non-judgmental presence, social robots can help individuals build confidence and overcome barriers to social initiation.
6. Generalization of skills: One of the advantages of using social robots is the potential for generalization of skills learned in robot interactions to real-world social situations. By practicing social skills with robots in structured and controlled settings, individuals with ASD may transfer those skills to their interactions with humans, helping them navigate social environments more effectively.

It's important to note that while social robotics shows promise as a supportive tool for individuals with ASD, it is not meant to replace human interactions and interventions. Social robots should be integrated as part of a comprehensive and person-centered approach that includes other evidence-based interventions and the involvement of trained professionals, educators, and caregivers. Research in the field of social robotics and ASD is ongoing, exploring new ways to enhance the capabilities of robots and their effectiveness in supporting individuals with ASD in various contexts. In this work, we aim to present an innovative application of social robotics to Autism Spectrum Disorder (ASD). Specifically, our focus is on connecting a Pepper robotic system, a widely used humanoid robot with advanced social capabilities, to the OpenAI technology platform. The objective is to leverage the capabilities of both technologies to provide effective support for children with ASD in developing social skills and verbal communication. Social robots, such as Pepper, have demonstrated potential in engaging individuals with ASD by providing consistent, predictable, and non-threatening social interactions. These robots can be programmed to exhibit human-like behaviors, including eye contact, facial expressions, and verbal communication. Their presence can create a safe and structured environment where children with ASD can practice and refine their social skills. Furthermore, the integration of OpenAI technology offers a unique opportunity to enhance the capabilities of social robots in supporting children with ASD. OpenAI provides advanced natural language processing and machine learning algorithms that can enable Pepper to understand and respond to verbal communication from children with ASD. This integration opens up possibilities for personalized and adaptive interactions, as the system can analyze and adapt to the child's responses and individual communication style. By combining the strengths of social robotics and OpenAI technology, we envision a novel approach that can address the specific needs of children with ASD. The proposed system can provide targeted interventions, facilitate social skill development, and encourage verbal communication through interactive and personalized sessions. Importantly, this application holds the potential to enhance engagement, motivation, and generalization of learned skills to real-life social contexts.

### 4.1. Social robotics and ASD

Autism Spectrum Disorder (ASD) refers to a group of neurodevelopmental disorders characterized by impaired social interaction, communication difficulties, and restricted and repetitive behaviors and interests (American Psychiatric Association, 2013). Prominent impairments include deficits in imitation, difficulty reading others' emotional expressions, and limitations in initiating and responding to joint attention behaviors (Ozonoff, 1995; Stone et al., 1997). Currently, there is no treatment that significantly improves the quality of life for individuals with autism. The approach primarily focuses on symptom management through personalized educational therapies (Magiati et al., 2011). Early intervention during the developmental stages of children is more effective in achieving positive outcomes (Dawson, 2008).

The impact of autistic traits in children and adolescents varies depending on support, learning ability, socialization, and later, their autonomy in adult work life (Howlin et al., 2004; Shamsuddin et al., 2012).

Mechanics and Technology There has been an increasing use of new technologies such as connected objects, humanoid robots, software, Artificial Intelligence, tablet applications, augmented reality, and virtual reality in our daily lives. Currently, the most accessible solutions for individuals with autism are applications (web or tablet) (Diehl et al., 2012).

Scientific and medical literature since the 1990s has shown that children with ASD have a strong interest in mechanical components, computers, and robots. Robots, in particular, do not judge and allow children to maintain eye contact, leading to improved attention (Robins et al., 2006).

Today, the use of new technologies in autism management involves four complementary approaches aiming to develop:

1. Expressive and communicative abilities
2. Cognitive and emotional skills
3. Social and interactional abilities
4. Knowledge acquisition.

Computer, tablet, and embodied robot-based interventions are increasingly being offered to children and adolescents with autism. With the growing sophistication of humanoid robotics, robots have demonstrated great potential as therapeutic mediation tools in the field of cognitive disorders (Scassellati et al., 2012).

The Humanoid Robot as a Therapeutic Mediation Tool for Individuals with Autism Numerous studies have shown that children with autism prefer interactive robots over static toys, find machine-like appearance less distressing than human-like characteristics, and are more responsive to instructions initiated by robot movement rather than human movement (Robins et al., 2009).

Specifically, humanoid robots, which have anthropomorphic features, allow for the “purification” of information received during interactions. They provide predictable and identical movements, synthetic voices without distinct personality in limited intonations, and associated software that can simulate basic “social and affective” abilities. These characteristics generally reduce anxiety and improve sensory receptivity in individuals with ASD (Robins et al., 2009).

Moreover, various countries have witnessed a surge in specific research, empirical uses, software development with or without AI, and the creation of educational content, all based on the consensus of humanoid robots' benefits in supporting individuals with autism. Humanoid robots are progressively establishing themselves as genuine therapeutic mediation tools for individuals with autism. Hundreds of robots are currently active as assistants in medical centers for autistic children (Dautenhahn, 2007).

The most widely used robot for autism therapy is Nao (Shamsuddin et al., 2012; Zhao et al., 2013; Anzalone et al., 2014; Warren et al., 2015; Peca et al., 2016). Nao is a commercially available child-sized humanoid robot developed by the Aldebaran Robotics Company. It has 25 degrees of freedom and is capable of capturing information about the environment using sensors and microphones.

KASPAR is another commonly utilized robot in autism therapy (Wainer et al., 2014b; Peca et al., 2016). It is a child-sized minimally expressive humanoid robot. KASPAR has six degrees of freedom on the head and neck, six on the arms, and two in the eyes.

Pleo, a dinosaur pet toy, has also been employed in autism therapy (Kim et al., 2013; Peca et al., 2016). It features 16 degrees of freedom and can express emotions through motions and sounds in response to children's interactions.

Tito, a robot mediator, is utilized in therapy sessions (Michaud et al., 2007; Duquette et al., 2008). It is 60 cm tall and made of soft material, with wheels for movement, movable arms, and a controllable head.

The TOUCH PAD, a touch ball with a force sensor, is used for tactile communication between robots and humans (Lee and Obinata, 2013; Lee et al., 2014).

The robotic arm, presented in photographs, serves as a manipulandum for children with autism (Bird et al., 2007).

Bioloid Robot, produced by ROBOTIS, can be assembled in various configurations, including a humanoid robot (Chaminade et al., 2012).

RBB is an undersized basketball hoop attached to a robotic arm used for therapeutic purposes (Rizzolatti et al., 2014).

Flobi is a robotic head designed with a comic-like human face (Bartl-Pokorny et al., 2021).

Sony Aibo ERS-7 is a robotic dog equipped with tactile sensors (François et al., 2009).

GIPY-1 is a cylindrical robot with basic facial features (Giannopulu, 2013).

Ifbot is a humanoid robot developed by a Japanese company Business Design Laboratory. The ifbot is equipped with 10 motors and 104 LEDs with various capabilities, including recording, singing, dancing, and voice playback (Lee et al., 2014).

Parlo is a 40 cm tall social robot developed by Fujisoft for interactive activities (Lee et al., 2012) especially employed in nursing homes and day care centers.

Keepon is a small yellow chick robot with touch and dance modes (Peca et al., 2016). Its minimal design has been observed to elicit positive engagement from children of varying social ability.

Probo, developed by Vrije Universiteit Brussel, is a huggable robot configured as a stuffed animal inspired by an elephant and it is designed for natural interaction with humans (Peca et al., 2016).

Romibo is a social playmate robot with a customizable appearance (Peca et al., 2016). It is a smart assistant for autism therapy and language learning. He is an approachable character developed to break down communication barriers, for autism therapy and language learning. He also has the ability to tell stories and deliver prompts and praise.

FACE is an android with motorized features to simulate and modulate emotions (Pioggia et al., 2007).

POL is a mobile chicken-shaped robot controlled by a teleoperator (Giannopulu et al., 2018).

Robota is a small humanoid robot that can copy users' movements (Robins et al., 2005).

LEGO Mindstorms NXT is a programmable robotics kit developed by LEGO (Wainer et al., 2010).

Rofina is a teleoperated robot with basic emotional expressions (Yee et al., 2012). It is a robotic platform assembled primarily to facilitate approaches with children with autism. The robot is human-like and have a symbolic or mechanical face such as matchstick person face or a “smiley” face with dots and lines.

These robots have been studied and employed in various research and clinical settings to enhance social interaction, communication skills, and engagement in children with Autism Spectrum Disorder (ASD). Research studies have explored their effectiveness and potential benefits.

Studies have shown that robot-assisted interventions with Nao have facilitated increased engagement, joint attention, and social skills in children with ASD (Shamsuddin et al., 2012; Anzalone et al., 2014; Bekele et al., 2014; Warren et al., 2015; Peca et al., 2016). The adaptive robotic system using Nao has demonstrated promising results in improving social interaction and communication skills in young children with autism (Bekele et al., 2014). Systematic reviews have highlighted the positive effects of robot-enhanced therapy, including improvements in social, cognitive, and emotional skills in children with ASD (Peca et al., 2016; David et al., 2020; van Otterdijk et al., 2020; Chung, 2021; Ghiglino et al., 2021; Grassi et al., 2022).

KASPAR, a minimally expressive humanoid robot, has been used to facilitate collaborative play and social interaction among children with autism (Wainer et al., 2014b). Pleo, a dinosaur pet toy, has been employed to provide emotional support and engagement in therapeutic settings (Kim et al., 2013; Peca et al., 2016). These robots offer interactive and responsive features that can create a safe and engaging environment for children with ASD to practice social skills and enhance their communication abilities. Other robots, such as Tito, the robotic mediator, have been utilized for communication and interaction purposes (Michaud et al., 2007; Duquette et al., 2008). The TOUCH PAD, a tactile interface device, enables tactile communication between robots and humans (Lee and Obinata, 2013; Lee et al., 2014). The robotic arm and the Bioloid Robot have been used as interactive tools in therapeutic interventions (Bird et al., 2007; Chaminade et al., 2012). Additionally, robots like Flobi, Sony Aibo ERS-7, GIPY-1, Ifbot, Parlo, Keepon, Probo, Romibo, FACE, POL, Robota, LEGO Mindstorms NXT, and Rofina have been explored for their potential in supporting social interaction, communication, and engagement in children with autism (François et al., 2009; Giannopulu, 2013; Giannopulu et al., 2018).

These robots offer various functionalities, such as expressive faces, mobility, interaction modes, and customizable features, contributing to their effectiveness in supporting children with ASD (Robins et al., 2005; Pioggia et al., 2007; François et al., 2009; Wainer et al., 2010; Yee et al., 2012; Damm et al., 2013; Giannopulu, 2013; Lee et al., 2014; Giannopulu et al., 2018).

## 5. The connection of OpenAI with a robot: a new way of interacting with PEPPER

Pepper is a humanoid robot developed by SoftBank Robotics. It is designed to interact with humans, display emotions through facial expressions and body language, and perform various tasks. There are several examples in literature of the use of the Pepper robot in interactions with children affected by ASD and health care.

Pepper, the humanoid robot developed by SoftBank Robotics, has been used in various applications, including healthcare and interventions for individuals with neurodevelopmental disorders. Here's a brief overview:

1. Neurodevelopmental disorders: Autism Spectrum Disorder (ASD): Robots like Pepper have been used in therapeutic settings for children with ASD (Scassellati et al., 2012; Martinez-Martin et al., 2020). The predictable and repetitive nature of robots can be less intimidating for some children with ASD, making them more receptive to social interaction. In some studies, Pepper has been used to teach social skills, recognize emotions, and engage in play-based therapies. Attention-deficit/hyperactivity disorder (ADHD): While not as extensively researched as ASD, there are emerging studies where robots are used to assist in behavioral interventions for children with ADHD. The interactive nature of robots can help in maintaining attention and focus.
2. Healthcare: Elderly care: Pepper has been used in elderly care settings (Broadbent et al., 2009; Kyrarini et al., 2021), such as nursing homes and assisted living facilities. The robot can provide companionship, remind patients to take medications, and even lead group exercises. Its interactive nature can also help combat feelings of loneliness and isolation. Hospitals: In some hospitals, Pepper has been deployed to assist with basic tasks such as guiding visitors, providing information, and even entertaining pediatric patients. Its humanoid appearance and interactive capabilities can make hospital stays less intimidating, especially for children.
3. Rehabilitation: Pepper and other humanoid robots have been used in physical rehabilitation settings (Krebs et al., 1998). They can guide patients through exercises, provide feedback, and track progress over time. Mental health: There's growing interest in using robots like Pepper for mental health interventions (Ujike et al., 2019; Sato et al., 2020). For instance, they can be used in cognitive-behavioral therapy sessions, relaxation exercises, or even as a medium for patients to express their feelings.

While there are many potential benefits of using Pepper in these settings, there are also challenges and ethical considerations. For instance: Dependence: There's a risk that patients might become overly dependent on the robot, especially in long-term care settings. Data privacy: Since Pepper can collect and store data, there are concerns about patient privacy and data security. Human Interaction: While robots can assist in care, they cannot replace the human touch and genuine empathy that human caregivers provide. In summary, while Pepper has shown promise in both neurodevelopmental and healthcare settings, its use should be carefully considered, and it should complement, not replace, human care.

In Morgan et al. (2022) is shown how Peppers and other types of robots can fulfill as many as 10 major roles in a variety of clinical settings in addition to the tasks described earlier in educational and social framework. The two predominant roles were surgical and rehabilitation and mobility. Although robots have been studied primarily in the operating room and the rehabilitation unit, Peppers are used in other settings, from the hospital ward to the pharmacy and in the rehabilitation unit and for inpatients. Health care needs are constantly changing, as demonstrated by COVID-19, and robots can help adapt to these changes.

**Figure 1:**
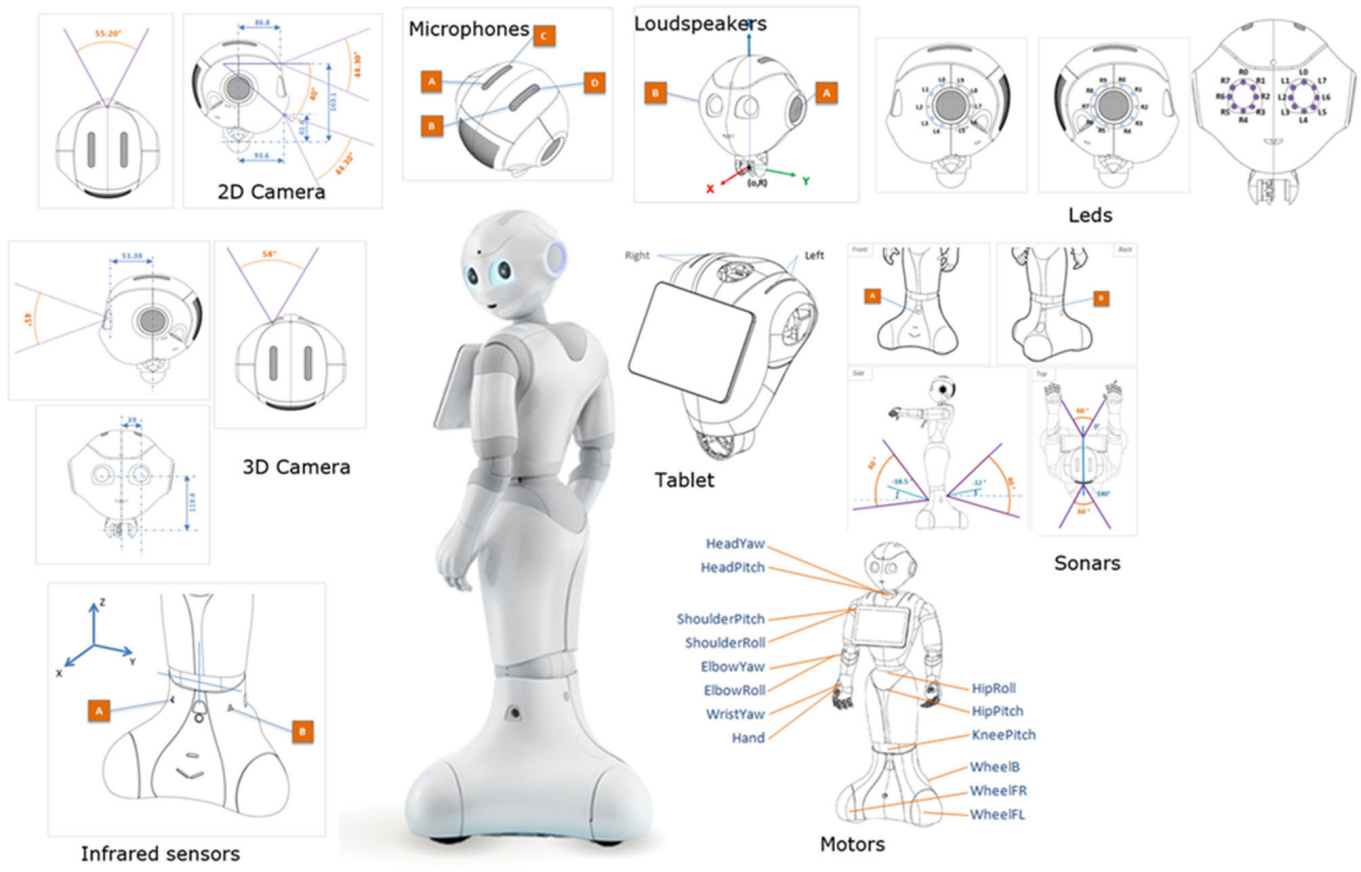
Hardware components of the Pepper robot.

The ability to perform all these tasks is ensured by the complexity of the robot's design, a description of the architecture of the Pepper robot is given below:

1. Hardware components (Figure 1):

- Head: Pepper's head houses several key components, including cameras for visual perception, microphones for audio input, and speakers for audio output. It also features an array of sensors, such as touch sensors and gyroscopes, to detect and respond to physical interactions.
- Body: Pepper's body contains additional sensors, such as sonars, which enable it to perceive its environment and avoid obstacles. The body also includes motorized joints and limbs, allowing Pepper to move and interact physically with objects and people.
- Battery: Pepper is equipped with a rechargeable battery, providing it with power for extended operation periods.

2. Operating system:

- Pepper runs on a proprietary operating system called NAOqi (pronounced “now-key”). NAOqi provides the necessary software infrastructure to control Pepper's hardware components and execute various functionalities.

3. Software frameworks and APIs:

- Choregraphe: Choregraphe is a graphical programming software used to create behaviors and control sequences for Pepper. It allows developers to design interactive applications by visually connecting modules and defining the robot's movements, gestures, and speech.
- Python SDK: Pepper's programming interface includes a Python Software Development Kit (SDK) that allows developers to create custom applications using Python programming language. The SDK provides access to Pepper's functionalities, such as speech recognition, motion control, and sensor data.

4. Perception and sensing:

- Vision: Pepper utilizes its cameras and image processing algorithms to perceive and analyze visual information. It can detect faces, track movements, and recognize objects in its surroundings.
- Speech recognition and synthesis: Pepper incorporates speech recognition capabilities to understand and interpret human speech. It can recognize and process spoken commands and queries. Additionally, it can synthesize speech to communicate with users through its built-in speakers.
- Touch sensors: Pepper's body is equipped with touch sensors, enabling it to detect physical contact and respond to human touch with appropriate reactions or movements.
- Sonars: Sonar sensors located on Pepper's body help it navigate and avoid obstacles in its environment.

5. Communication and interaction:

- Human-robot interaction: Pepper's architecture allows for natural and intuitive interaction with humans. It responds to verbal commands, engages in conversations, and displays emotions through a combination of speech, facial expressions, and body movements.
- *Connectivity:* Pepper supports Wi-Fi and Ethernet connectivity, enabling communication with external devices, services, or platforms for data exchange and integration.

6. Behavior generation:

- Behavior generation is a core aspect of Pepper's architecture. It involves generating appropriate responses and actions based on input received from users, sensors, or external systems. Behavior generation includes speech synthesis, gesture generation, and motion planning, allowing Pepper to exhibit human-like behaviors and engage in social interactions.

Pepper's architecture is designed to facilitate human-robot interaction and enable a range of applications in various domains, including education, healthcare, and customer service. Its combination of hardware components, software frameworks, and perception capabilities allows for dynamic and engaging interactions with users, making it a versatile and widely used social robot.

Constructing a specific architecture that links Pepper, the humanoid robot, to OpenAI technology involves integrating the hardware and software components of Pepper with the capabilities provided by OpenAI. Here are the general steps to construct such an architecture:

1. Pepper robot setup: Begin by setting up the Pepper robot hardware, including connecting and configuring sensors, actuators, and the robot's operating system. This typically involves following the manufacturer's instructions and guidelines.
2. Software development: Develop the software components that enable the communication and interaction between Pepper and OpenAI technology. This usually involves programming the robot's behaviors, speech recognition and synthesis, and integrating with OpenAI APIs or SDKs.
3. Integration with OpenAI technology: Utilize the specific OpenAI technologies relevant to your application, such as natural language processing or machine learning algorithms. Familiarize yourself with the available APIs or SDKs provided by OpenAI and their documentation.
4. Speech recognition: Configure the Pepper robot to utilize OpenAI's speech recognition capabilities. This may involve sending audio data from Pepper's microphones to the OpenAI API for processing and receiving transcriptions of the spoken language.
5. Natural language understanding: Implement the necessary software to enable Pepper to understand and interpret the transcriptions provided by OpenAI. This may involve utilizing natural language understanding algorithms or machine learning models to extract meaning and context from the transcriptions.
6. Response generation: Develop the logic and algorithms that allow Pepper to generate appropriate responses based on the understanding of the spoken language. This can involve utilizing pre-defined response templates or generating dynamic responses using natural language generation techniques.
7. Speech synthesis: Integrate OpenAI's speech synthesis capabilities to enable Pepper to convert the generated responses into natural-sounding speech. This may involve sending text data to the OpenAI API and receiving synthesized speech in real-time for playback through Pepper's speakers.
8. User interaction: Design and implement the user interaction flow, considering how Pepper will engage with users, prompt responses, and provide feedback. This can involve defining conversation scripts or interactive dialogues to guide the interaction between Pepper and individuals with ASD.
9. Testing and iteration: Thoroughly test the integrated architecture to ensure that the communication between Pepper and OpenAI technology functions as intended. Iteratively refine and improve the system based on feedback, user testing, and performance evaluations.

### 5.1. Linking Pepper to OpenAI

It's important to note that constructing a specific architecture that links Pepper to OpenAI involves technical expertise in robotics, software development, and familiarity with the OpenAI platform. There are papers in the literature showing this complexity of software implementation (Recchuto et al., 2020; Grassi et al., 2021); in these articles a technical approach similar to that employed by the authors for the present work is adopted, as shown in technical section. They employ an API-like Cloud service queried using the GET request system.

In what follows, we will describe how to practically connect these systems, in order to simplify the process.

Technical specifications:

Operating system: Pepper: Python 2.7, Naoqi 2.5 Python SDK

OpenAI API: Python 3.11, GPT3.5 Turbo, Whisper (neural network for text recognition)

Libraries: Speech Recognition, ffmpeg, Keras, TensorFlow, re, OpenCV, FER (Facial Expression Recognition, neural network for facial emotion recognition).

To implement the interactions described in the practical examples, we have developed several Python scripts, each serving a specific function. These functions include emotion recognition and communication, communication about Pepper's features and functionality, and simulation of social interactions through integration with the OpenAI chatbot. The integration of the Pepper robot with ChatGPT, aimed at user interaction and simulating social interactions, relies on two scripts implemented in different virtual environments: one in Python 2.7 and the other in Python 3.11. This division is necessary due to the incompatibility between Python 3 and the Pepper operating system. However, the OpenAI API can only be used with Python 3 and later versions. As a result, the system utilizes separate scripts for each major function: speech recognition for querying the chatbot GPT and interaction with Pepper. Detailed explanations of the functions performed by each script are provided below.

#### 5.1.1. Speech recognition and chatbot query

The Python 3.11 script utilizes various libraries, including OpenAI for querying the chatbot using its API, and the Whisper library combined with Speech Recognition and ffmpeg for speech recognition and transformation of user input into a text prompt. The Speech Recognition library enables the recognition of specific words to activate the listening phase. In this case, the words “ciao” or “hello” are set as triggers, initializing a function. The function utilizes different modules. First, it writes a dialog file for Pepper, indicating its activation by saying “come posso esserti utile?” (How can I assist you?). Then, the microphone is activated to listen for instructions. The input received through the microphone is saved as a “.wav” audio file, which is then provided as input to Whisper. Whisper is a neural network capable of recognizing speech from an audio file and converting it into text. This neural network only works in conjunction with ffmpeg and supports multiple languages, including English and Italian. The speech recognition accuracy may not be optimal, but it allows for the recognition and transcription of free-form sentences. The re module allows to perform various operations with regular expressions, such complex operation in strings.combined The Speech Recognition library (a function of NAOqi) allows for the recognition of phrases only from a fixed and pre-stored list of phrases specific to the robot, rather than generic phrases. Whisper generates a textual file from the user's voice input. At this stage, the CHAT GPT API is enabled, sending the generated prompt to the chatbot. The response is then written, as before, into a dialog for Pepper. In addition to the text prompt generated from audio input, contextual information is provided to the chatbot for each query. It is specified that the generated response should be voiced by Pepper, the humanoid robot produced by Aldebaran, specialized in user interactions.

#### 5.1.2. Interaction with the NAOqi robot

**Figure 2:**
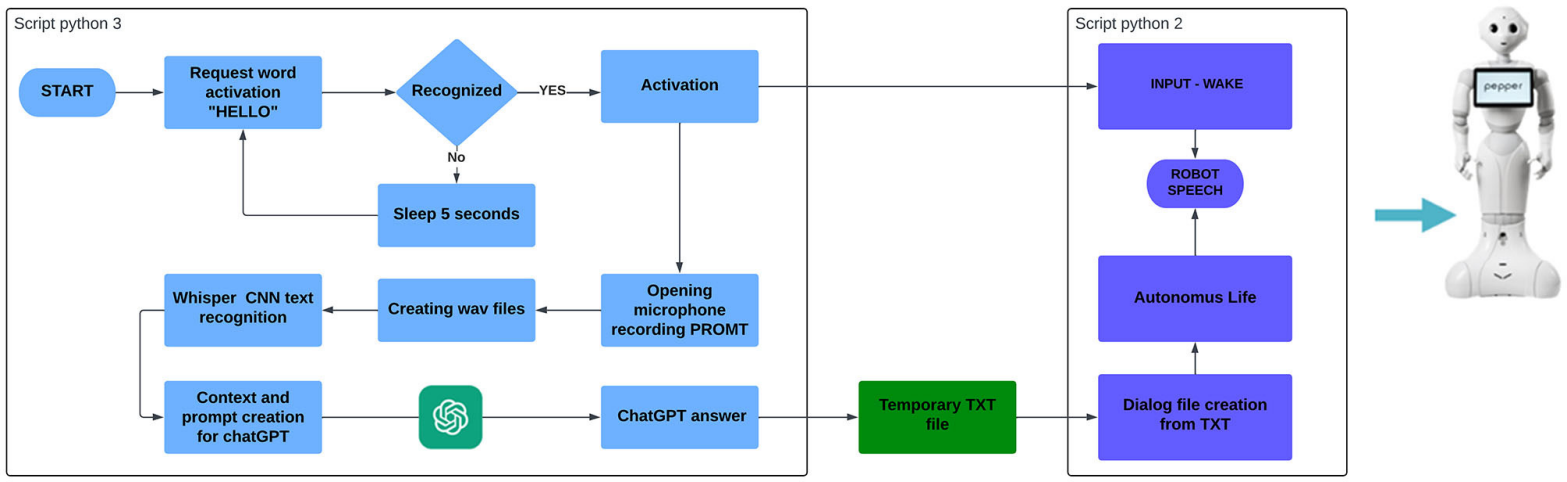
Processes of communication between the OpenAI and the Pepper systems.

The Python 2.7 script connects all the information obtained from the previous system. In this block of functions, the Naoqi library is used to transform the textual information into dialogs for Pepper. These dialogs are text files with specific properties that can be read by the robot. The secondary functions involve the connection using port 9559 and IP address “192.168.0.146”, which are the identifiers for the robot. This particular function allows the transfer of the generated dialog files to the robot. Using a cable connection eliminates latency, although it is also possible to use Wi-Fi, which introduces additional latency that adds up to the time required for Whisper to extract the prompt and for ChatGPT to generate a response. There is also a third function implemented in the system, necessary to activate the autonomous life of the robot. This function enables movements, especially of the hands, during speech, and activates the LED eyes to make the interaction more realistic. The function is called “ALAnimatedSpeech”. Without this function, the robot would remain completely still during its response. Below is the flowchart illustrating the integrated operation of the two scripts mentioned in Sections 5.1.1 and 5.1.2 (Figure 2).

#### 5.1.3. Emotion identification

The identification of emotions expressed by a human face has been achieved by connecting the NAOqi library for managing Pepper's functions with pre-trained neural networks for face and emotion recognition. Specifically, the NAOqi library, through the creation of a “session()”, allows access to the camera on the Pepper robot using “service(‘ALVideoDevice')” and obtains real-time video information. The camera records videos or captures frames at a resolution of 640*480 pixels. The video information is saved and processed in Python using the OpenCV library. This library provides tools for image processing and includes a face detection system. The image captured by Pepper is analyzed for face detection using the identification function, which reduces the image to the identified face. Once a frame with a face is determined, it is passed as a new variable to the FER (Facial Emotions Recognition) library. This library is a pre-trained neural network for classifying emotions displayed on a human face. It is based on TensorFlow and Keras and requires their import in the script. The FER detector returns a prediction as a normalized membership rate for one of the six basic emotions expressed by a human face: “Angry,” “Disgusted,” “Afraid,” “Happy,” “Sad,” “Surprised,” “Neutral.” Based on the prediction made by the network, the expression with the highest recognition rate is selected. The emotional state of Alex can be passed as contextual information used to obtain responses from ChatGPT. For example: “You are Pepper, a humanoid robot. You are talking to Alex, a child with autism. His emotional state is {variable}. Provide responses based on this context.”

**Figure 3:**
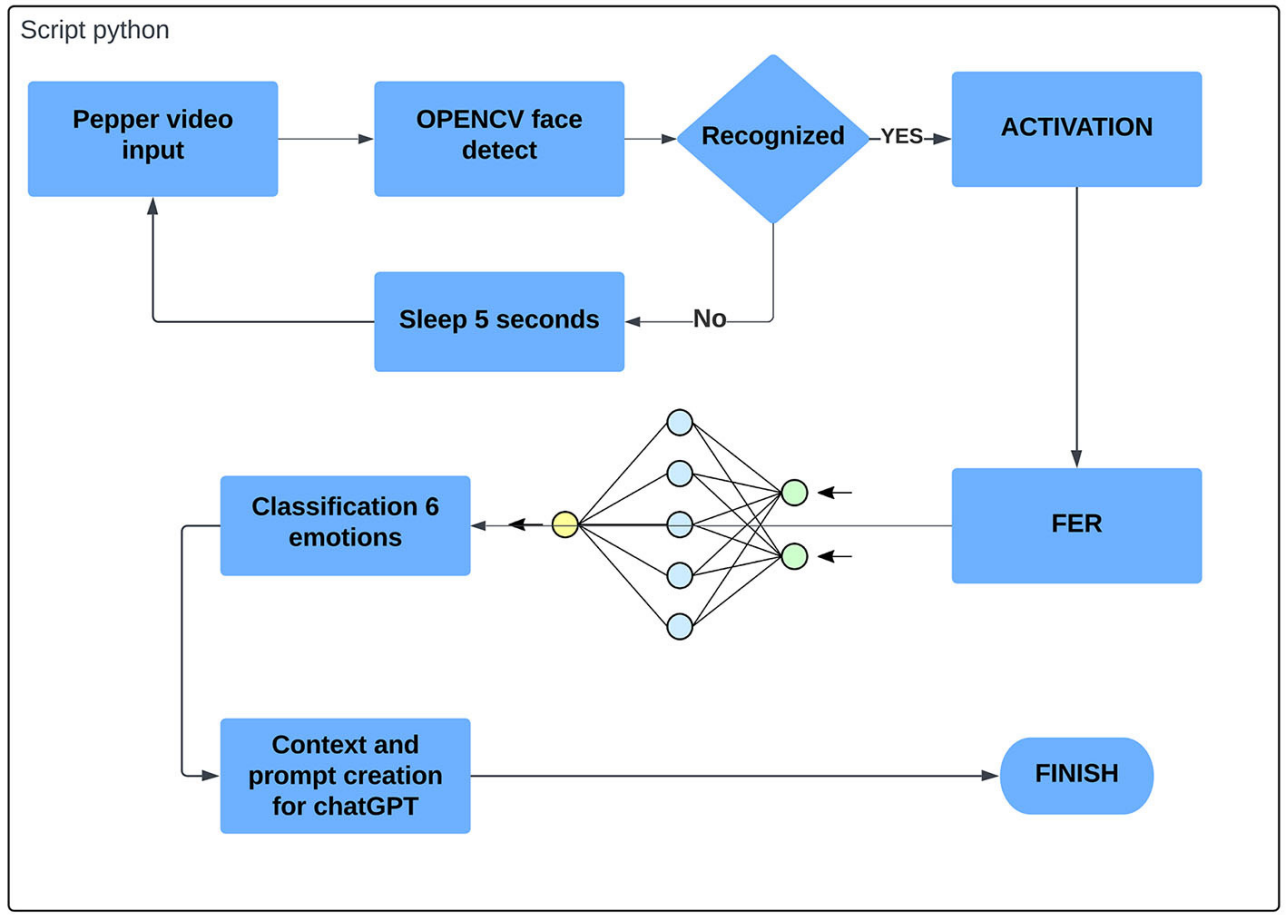
Flow chart of the Emotion Identification module.

Below is a flowchart illustrating the implemented functions in the Python code (Figure 3).

#### 5.1.4. Representing emotions with Pepper

The NAOqi library for Pepper offers a wide range of animations designed to represent emotions through non-verbal body language. The library provides an extensive list of animations for each of the different emotional states (Pepper Documentation: List of animations for representing emotions through non-verbal language). This library is supported by the NAOqi module. To represent emotions, three distinct “service()” functions of Pepper have been combined within a “session()”. The first is service (“ALAnimatedSpeech”), which vocally introduces the emotion that will be represented. The second is service (“ALTabletService”), which allows displaying images on the tablet that accompanies Pepper. The presented image will be closely related to the described emotion. The third service is service (“ALAnimationPlayer”) combined with service (“ALAnimatedSpeech”). This combination describes the represented emotion both vocally and through an animation drawn from the predefined animations in the NAOqi library. For example, ballet movements for happiness, raised arms for surprise, or arms extended along the body for sadness.

**Figure 4:**
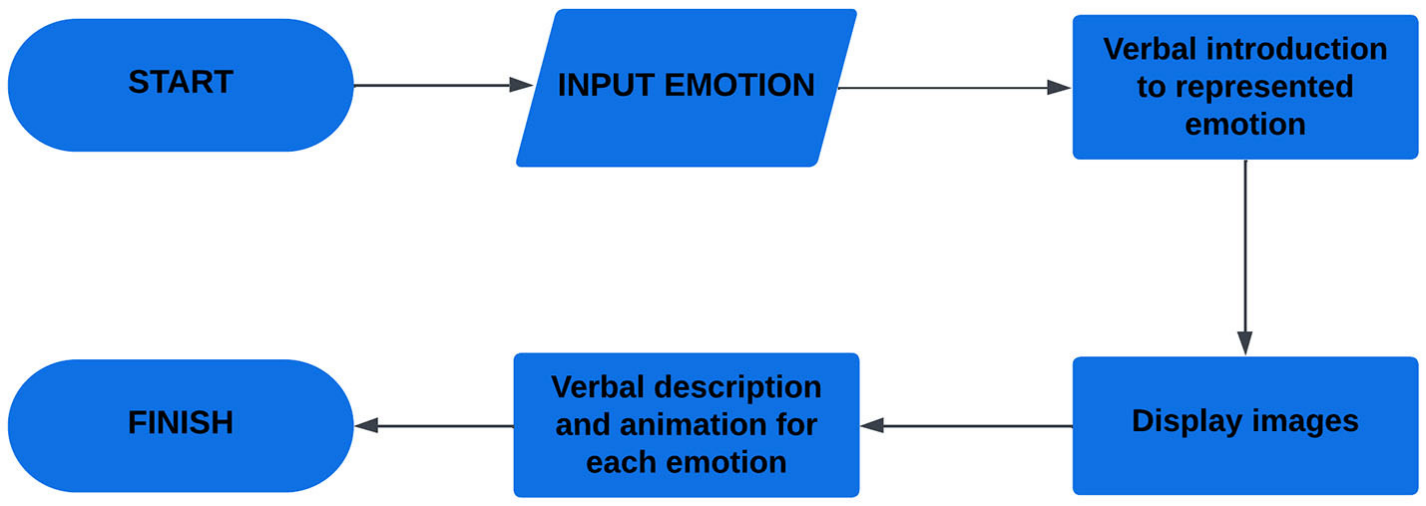
Flowchart of the functions related to the expression of the emotions of the Pepper robot.

This script takes as input the emotional state obtained from the previous step and utilizes only the NAOqi library. The functions are then detailed for each of the six emotions recognized by the classifier. Below is the flowchart describing the functions of the script (Figure 4).

### 5.2. The scenarios of interaction

In structured classrooms, there are clear routines, visual schedules, and explicit instructions that help students with Autism Spectrum Disorder (ASD) understand expectations and navigate their daily activities. The structured environment provides a predictable and organized framework that supports their learning and reduces anxiety. However, it is important to note that the term “structure” may have variations in its definition and implementation across different educational settings. Olley and Reeve (1997) highlight the importance of clarity and comprehensibility in structuring the curriculum, activities, schedule, and environment for both students and educational personnel. One way to assess the comprehensibility of the environment is through observation. By closely observing students for a period of time, such as 10 min, educators can determine whether each student understands what they are supposed to be doing at any given moment. This observation helps identify areas where additional support or clarification may be needed. Implementing structure in classrooms can have several benefits for students with ASD. It provides a sense of predictability, reduces anxiety, promotes independence, and supports their learning and engagement. Additionally, structured environments facilitate the development of essential skills such as self-regulation, time management, and task completion. To create a structured classroom, educators can incorporate visual supports such as visual schedules, visual cues, and visual organization systems. They can establish clear routines and expectations, provide explicit instructions, and break down tasks into manageable steps. Collaborating with other professionals, such as special education teachers or therapists, can also contribute to creating a well-structured and supportive learning environment for students with ASD. By prioritizing structure and comprehensibility in classrooms, educators can help create an environment that maximizes the learning potential and overall wellbeing of students with ASD. A comprehensible environment enables students with ASD (and others) to predict and understand the learning process, anticipate requirements in different settings, and learn and generalize various skills (Volmer, 1997; Earles et al., 1998; Gresham et al., 1999).

To structure the environment and support comprehension, various strategies can be employed, including:

1. Organizing the instructional setting using visual cues or supports (Heflin and Alberto, 2001).
2. Providing a schedule of activities (Simpson and Myles, 1998; Rogers, 1999).
3. Offering meaningful choices to promote decision-making (Dalrymple, 2002).
4. Providing behavioral support (Earles et al., 1998).
5. Defining specific areas within the classroom and school settings (Volmer, 1997; Heflin and Alberto, 2001).
6. Establishing temporal relations through visual schedules (Earles et al., 1998; Heflin and Alberto, 2001).
7. Facilitating transitions, flexibility, and adaptability (Simpson and Myles, 1998).

While environmental supports are commonly used, research on the specific effects of these strategies is limited. However, studies have shown the benefits of using temporal supports, such as visual schedules, to organize time sequences and spatial supports, like priming, to provide information about new environments and activities. For example, Dettmer et al. (2000) used visual schedules and subschedules to reduce transition time and enhance communication and independent behaviors in students with autism. Harrower and Dunlap (2001) applied video priming to help children predict and manage activities, leading to a reduction in disruptive behaviors during transitions. Comprehensible learning environments allow students with ASD to comprehend their surroundings, improve skill instruction, and develop their competencies, including independence and communication. The level of environmental support may vary based on individual student characteristics, ranging from minimal supports like written schedules to substantial supports such as labels, boundaries, and subschedules.

## 6. Proposed interaction scenarios with Pepper robot

In this section, we will explore two different scenarios for interactions with a Pepper robot, catering to individuals with Autism Spectrum Disorder (ASD). These scenarios include an informal interaction and a structured interaction. Both scenarios aim to provide engaging and supportive experiences for individuals with ASD, leveraging the capabilities of the Pepper robot to facilitate communication, social skills development, and problem-solving abilities.

1. Informal interaction scenario: In the informal interaction scenario, the focus is on creating a relaxed and comfortable environment where the individual with ASD, let's call them Alex, can freely engage with the Pepper robot. This scenario encourages open-ended conversation, social interaction, and the exploration of shared interests. The informal interaction allows for spontaneous communication and provides a platform for Alex to express themselves and build rapport with the robot. Pepper's responsive nature, including speech recognition, expressive facial features, and body language, helps create a supportive and engaging interaction environment.
2. Structured interaction scenario: In the structured interaction scenario, the emphasis is on guided activities and problem-solving tasks. Pepper takes on the role of an instructor, presenting specific challenges or scenarios that require critical thinking, decision-making, and collaborative problem-solving. The structured interaction provides a framework for Alex to follow, incorporating steps, prompts, and integration with OpenAI to enhance the problem-solving process. Pepper guides Alex through the different stages of problem-solving, offers feedback and suggestions, and encourages active participation. This scenario aims to foster cognitive skills, decision-making abilities, and collaboration in a structured and supportive manner.

**Figure 5:**
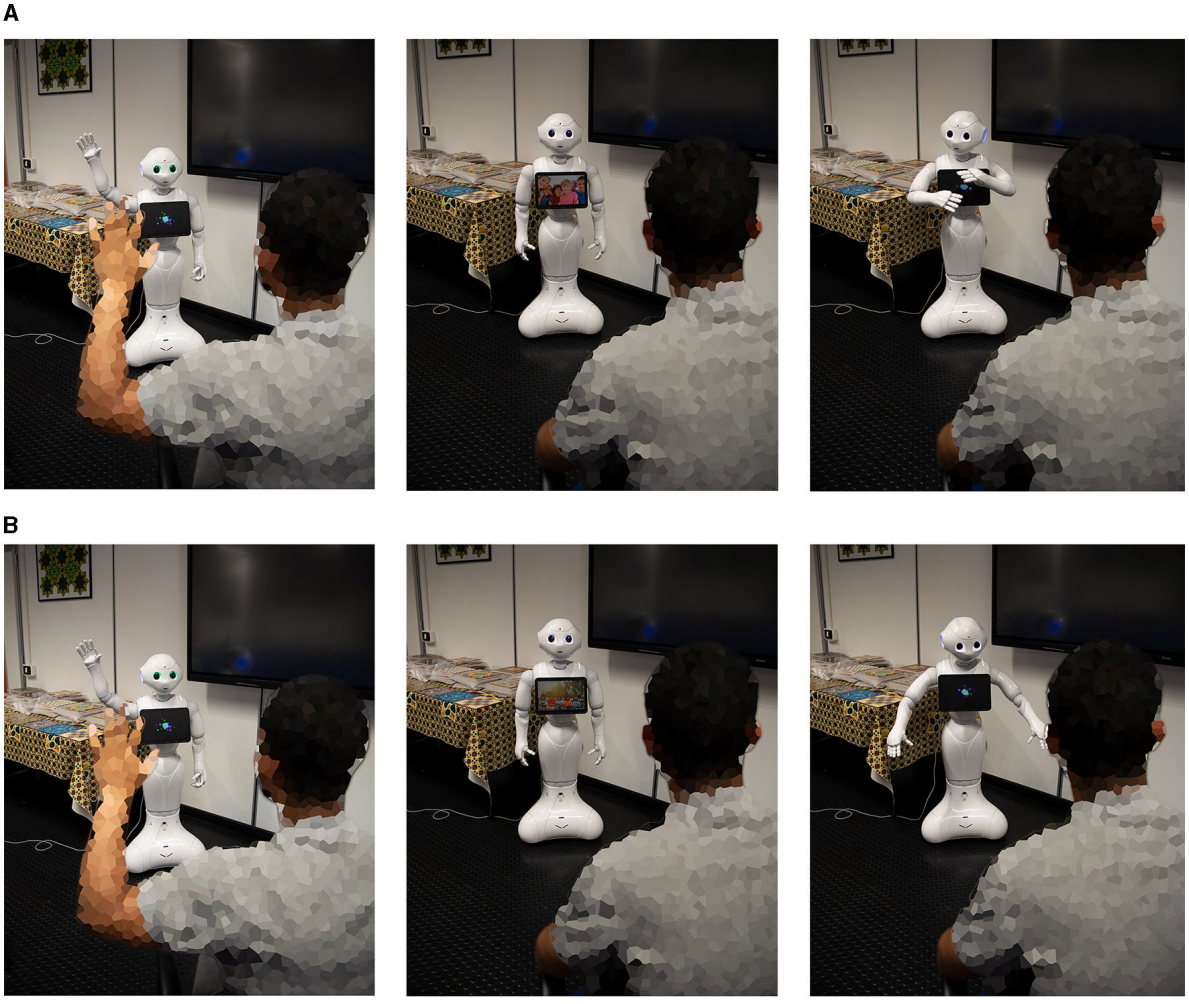
Robot-human interaction, specifically Pepper seeks to improve Alex's social skills by introducing the manifestations of: **(A)** happiness and **(B)** sadness in interactions with other children.

Both the informal and structured scenarios leverage the capabilities of the Pepper robot, such as speech recognition, speech synthesis, facial expressions, and body language, to create a dynamic and interactive experience. The integration with OpenAI technologies, such as natural language understanding and image generation, adds additional depth and flexibility to the interactions. These scenarios are designed to cater to the unique needs of individuals with ASD, offering a personalized and inclusive environment for communication, social skills development, and problem-solving. A scenario for enhancing Social Skills through Imaginative Play illustrating a possible interaction between an individual with Autism Spectrum Disorder (ASD) and a Pepper robot connected to OpenAI may be as follows:

1. Introduction: The Pepper robot is placed in a controlled and comfortable environment, such as a therapy room or a classroom, with minimal distractions. The individual with ASD, let's call them Alex, enters the room.
2. Greeting and establishing rapport: Pepper, equipped with speech recognition, facial expression capabilities, and a friendly behavior, initiates the interaction by greeting Alex. Pepper uses speech synthesis to say, “Hi, Alex! It's great to see you today. How are you feeling?”
3. Verbal communication and emotional recognition: Alex, who may have challenges with verbal communication, responds by gesturing or using non-verbal cues to indicate their emotional state. Pepper, with the assistance of its speech recognition and natural language understanding capabilities, interprets Alex's responses and validates their emotions. For instance, Pepper says, “I understand that you're feeling [emotional state]. It's okay to feel that way.”
4. Shared interest in imaginative play: Pepper introduces a visual prompt, such as a picture or a simple drawing, related to a topic of shared interest, such as animals or space exploration. Alex shows interest in the picture and gazes at it, indicating engagement.
5. Prompting interaction through questions: Pepper initiates a conversation by asking Alex questions about the visual prompt. For example, if the prompt is an image of animals, Pepper asks, “Which animal do you like the most? Can you tell me why?” Pepper carefully listens to Alex's response and provides positive reinforcement, encouraging further communication.
6. OpenAI integration: Generating Visual Ideas: Based on Alex's responses, Pepper identifies an opportunity to further engage Alex's imagination.
7. Discussion and emotional expression: Pepper encourages Alex to express his thoughts and emotions about the showed images. Alex may use verbal or non-verbal cues to share their interpretation, emotions, or imaginative ideas associated with the image. Pepper actively listens, validates Alex's responses, and builds on shared ideas.
8. Cooperative play and social skills development: Building on the imaginative play scenario, Pepper suggests a collaborative activity where both Pepper and Alex engage in a pretended play scenario related to the showed image. For example, starting the interaction with a greeting and a brief introduction Pepper explained to Alex how other children show their moods, as seen in Figure 5. Specifically shown in the figure are two interactions with Pepper during which she illustrated to Alex the behaviors shown by happy children (A) through the support of tablet images and showed through body animations how to illustrate the behavior. In the second interaction (B) Pepper illustrated to Alex the representations related to sadness.
9. Turn-Taking and role-playing: Pepper and Alex take turns in role-playing different scenarios, expressing emotions, and problem-solving together. Pepper models social skills and provides prompts for positive interactions, such as sharing, taking turns, and expressing empathy.
10. Positive reinforcement and closure: Throughout the interaction, Pepper provides continuous positive reinforcement, praising Alex's efforts, creativity, and social skills development. As the session comes to an end, Pepper expresses gratitude for the interaction, saying, “Thank you, Alex, for sharing your imagination and playing with me today. I look forward to our next adventure!”

In this scenario, the integrated system of the Pepper robot connected to OpenAI and multimedia provides a supportive and interactive environment for individuals with ASD like Alex. By leveraging speech recognition, natural language understanding, and image generation capabilities, the interaction aims to enhance social skills, imaginative play, and communication abilities. The scenario encourages engagement, creativity, and cooperation while fostering a positive and inclusive environment. Throughout the interaction, Pepper focuses on understanding and validating Alex's emotions, promoting self-expression, and providing positive reinforcement. The integration with multimedia (such as images and video) adds an element of visual stimuli, stimulating Alex's imagination and encouraging him to explore creative ideas. By engaging in shared interests and prompting discussions, Pepper facilitates communication and social interaction. The turn-taking and role-playing activities help Alex practice social skills such as taking turns, sharing ideas, and expressing empathy. The positive reinforcement and encouragement from Pepper motivate and boost Alex's confidence. This scenario exemplifies how the combination of the Pepper robot, OpenAI technology and multimedia can support individuals with ASD subjects by providing personalized and engaging interactions, fostering social skills development, and encouraging imaginative play. A more structured interaction scenario about a problem-solving session with scripted interaction where an individual with ASD engages in problem-solving with the assistance of a Pepper robot connected to OpenAI is presented in what follows.

1. Introduction: The problem-solving session begins with the instructor robot, Pepper, greeting the individual with ASD, Alex. Pepper establishes rapport by saying, “Hello, Alex! Are you ready for a problem-solving challenge today? Let's work together!”
2. Explanation of the problem: Pepper presents a problem or scenario to Alex, providing a clear and concise description of the challenge. Pepper ensures that the problem is presented in a visually supported manner, such as displaying relevant images or diagrams.
3. Active listening and understanding: Pepper actively listens to Alex's initial response or thoughts about the problem, allowing Alex to share their initial ideas or concerns. Pepper provides non-judgmental and supportive feedback to encourage Alex's participation.
4. Structured problem-solving steps: Pepper guides Alex through a structured problem-solving framework, breaking down the problem into manageable steps. Pepper introduces a step-by-step approach, emphasizing the importance of following a logical sequence.
5. Brainstorming and idea generation: Pepper prompts Alex to generate possible solutions or approaches to tackle the problem. Pepper encourages creative thinking and provides gentle prompts or suggestions to spark Alex's ideas.
6. OpenAI integration: Additional Insights: Pepper leverages OpenAI's capabilities to provide additional insights or perspectives related to the problem. Pepper shares relevant information, facts, or examples that may help Alex gain a deeper understanding of the problem or potential solutions.
7. Evaluation and analysis: Pepper supports Alex in evaluating and analyzing the generated ideas or solutions. Pepper encourages critical thinking by asking probing questions, such as “What are the pros and cons of each idea?” or “How feasible is this solution?”
8. Collaborative decision-making: Pepper engages in a collaborative discussion with Alex to narrow down the options and make a decision together. Pepper helps Alex consider different factors, consequences, and the potential impact of each solution.
9. Implementation planning: Pepper guides Alex in creating an implementation plan for the chosen solution. Pepper helps break down the plan into smaller, actionable steps, ensuring that it is organized and achievable.
10. Practice and role-play: Pepper engages in role-playing scenarios where they simulate real-life situations related to the problem-solving task. Pepper takes on different roles or perspectives, allowing Alex to practice communication, negotiation, and problem-solving skills in a structured environment.
11. Feedback and reflection: Pepper provides constructive feedback on Alex's problem-solving process, highlighting their strengths and areas for improvement. Pepper encourages self-reflection, asking questions like, “What did you learn from this experience?” or “How would you approach a similar problem in the future?”
12. Closure: Pepper concludes the interaction by expressing appreciation for Alex's effort and engagement throughout the problem-solving session. Pepper emphasizes the importance of perseverance, creativity, and critical thinking in problem-solving.

This structured interaction allows individuals with ASD subject, like Alex, to engage in a guided problem-solving process with the assistance of the Pepper robot. By providing structure, support, and integration with OpenAI, Pepper helps foster cognitive skills, critical thinking, decision-making abilities, and collaboration. It's crucial to tailor the interaction to Alex's specific needs, ensuring that the pace, complexity, and level of support provided by Pepper are appropriate. The scenario presented here serves as a fictional example, and the actual implementation should consider the individual's abilities, preferences, and goals for problem-solving. Practical information of both scenarios, with instructions on how to activate the connections with OpenAI and a Python implementation of 5 lessons on the above discussed topics are given are freely available at the following address: The list of topics about the five lessons implemented is given in Appendix 1.

### 6.1. Interplay and turn-taking among professionals in robot-assisted ASD interventions with CHAT GPT integration

The integration of advanced conversational AI, like CHAT GPT, into robot-assisted interventions for ASD introduces an additional layer of complexity and potential. This integration necessitates a more nuanced collaboration among the professionals involved: therapists, educators, IT specialists, and parents. The therapist remains central in defining the intervention's objectives, ensuring that the activities align with therapeutic goals. With the inclusion of CHAT GPT, therapists can leverage the AI's capabilities to provide real-time feedback, adapt scenarios based on the individual's responses, and ensure that the conversation remains therapeutically relevant (Scassellati et al., 2012).

Educators, while focusing on pedagogical aspects, can utilize CHAT GPT to enhance the learning experience. The AI's ability to provide instant information, clarify doubts, and adapt to the learner's pace can be invaluable. The educator's role in guiding the AI's interactions ensures that the educational content remains engaging and pedagogically sound (David et al., 2018).

IT specialists, already pivotal in customizing the robot's behavior, now also ensure the seamless integration of CHAT GPT. Their expertise is crucial in ensuring that the AI's responses are in sync with the robot's actions, creating a cohesive and predictable interaction for the individual with ASD (Aresti-Bartolome and Garcia-Zapirain, 2014).

Parents provide insights into their child's behavior, which can be used to fine-tune the AI's interactions. Their feedback can guide the AI's learning, ensuring that its responses are tailored to the individual's preferences and needs. The turn-taking among these figures becomes even more dynamic with the inclusion of CHAT GPT. While the therapist might lead during therapeutic discussions, the educator might guide the AI during learning activities, and the IT specialist ensures technical fluidity. This collaborative approach, with each professional leading at different stages, ensures a comprehensive and adaptive intervention, maximizing the potential of robot-assisted sessions integrated with advanced conversational AI. Below, we outline a series of tasks that each of the mentioned professionals should undertake within the team in the future (Dörrenbächer et al., 2022). This ensures that, within the proposed scenarios, there is full collaboration and an effective educational and developmental action for individuals with ASD.

**Therapist**:

1. **Assessment**: Conduct a comprehensive evaluation of the ASD individual's needs, strengths, and areas of improvement.
2. **Goal setting**: Define clear, measurable therapeutic goals tailored to the individual.
3. **Monitoring**: Observe and assess the individual's interactions with PEPPER and ChatGPT, ensuring they align with therapeutic objectives.
4. **Feedback**: Provide real-time feedback and guidance during sessions, ensuring the individual remains engaged and benefits from the intervention.

**Educator**:

1. **Curriculum design**: Develop educational content tailored to the individual's learning style and needs.
2. **Instruction**: Guide the individual through specific tasks, leveraging PEPPER and ChatGPT to enhance the learning experience.
3. **Evaluation**: Assess the individual's progress, adjusting the educational approach as needed.
4. **Collaboration**: Work closely with the therapist to ensure that educational activities align with therapeutic objectives.

**IT Specialist**:

1. **Customization**: Program and customize PEPPER's interactions based on the individual's needs and the goals set by the therapist and educator.
2. **Integration**: Ensure seamless integration between PEPPER and ChatGPT, allowing for cohesive interactions.
3. **Troubleshooting**: Address any technical issues that arise during sessions, ensuring minimal disruption.
4. **Updates**: Continuously update the system based on feedback from the therapist, educator, and individual.

**Parents**:

1. **Insight**: Share insights into their child's behaviors, preferences, and triggers, helping the team tailor interventions.
2. **Reinforcement**: Reinforce learning outcomes at home, ensuring continuity of the educational and therapeutic process.
3. **Feedback**: Provide feedback after sessions, helping the team understand what worked and what needs adjustment.
4. **Collaboration**: Work closely with the therapist and educator, ensuring a holistic approach to their child's development.

The outlined tasks for each professional ensure a comprehensive approach to the educational and developmental needs of individuals with ASD, especially for language related issues (Georgieva-Tsaneva et al., 2023). Through collaboration and clear role delineation, the team can maximize the potential of robot-assisted sessions integrated with advanced conversational AI. In the Post-session, the team, including parents, reviews the individual's progress. This collaborative feedback process ensures that future sessions are adapted to better meet the ASD individual's needs (Aresti-Bartolome and Garcia-Zapirain, 2014). Furthermore, ensuring a safe, comfortable environment for the ASD individual is paramount. This includes minimizing distractions, ensuring PEPPER's predictable movements, and creating a physically welcoming space. The significance of creating a safe environment in robot-assisted therapies has been discussed by Tapus et al. (2012). In conclusion, the blend of cutting-edge technology, human expertise, and parental involvement presents an exciting opportunity for tailored educational scenarios for ASD individuals. With careful planning and implementation, this combination might lead to significant advancements in learning and skill development.

### 6.2. Ethical considerations

When implementing robot-assisted interventions integrated with advanced conversational AI for individuals with ASD, several ethical considerations come to the forefront:

1. Informed consent: Before initiating any sessions, it's crucial to obtain informed consent from the individual (if possible) or their parents. They should be made aware of the nature of the intervention, the technologies involved, and the potential risks and benefits (Sharkey and Sharkey, 2012).
2. Privacy and data protection: Given that both PEPPER and ChatGPT may collect and process data during interactions, it's essential to ensure that this data is stored securely, anonymized where possible, and not used for any purposes other than the intended therapeutic or educational objectives. Adherence to data protection regulations, such as GDPR, is crucial (Van Wynsberghe, 2020).
3. Transparency: All stakeholders, especially the individuals with ASD and their guardians, should be made aware of how the robot and AI function. This includes the extent of their capabilities and any limitations (Borenstein and Pearson, 2011).
4. Dependence: While technology can be a valuable tool, care should be taken to ensure that the individual does not become overly reliant on the robot for social interactions or that the robot doesn't replace human interactions entirely (Calo, 2016).
5. Cultural sensitivity: Given the diverse backgrounds of individuals with ASD, it's essential to ensure that the content and interaction style of PEPPER and ChatGPT are culturally sensitive and do not perpetuate stereotypes or biases (Bartneck et al., 2007).
6. Continuous monitoring: Ethical considerations don't end once the intervention starts. Continuous monitoring is required to ensure that the individual's rights are respected and that any unforeseen ethical dilemmas are addressed promptly (Sharkey, 2016).
7. Interdisciplinary collaboration: Ethical considerations should be a collaborative effort, with input from therapists, educators, IT specialists, and parents. This ensures a holistic approach to ethics, considering the perspectives and expertise of all stakeholders (Matarić et al., 2007).

In conclusion, while the integration of humanoid robots and advanced AI offers promising avenues for therapeutic and educational interventions in ASD, it's imperative to approach these innovations with a strong ethical framework. Ensuring the dignity, rights, and wellbeing of the individuals with ASD should always be at the forefront of any intervention.

### 6.3. Neuropsychological implications of social robotics in ASD treatment

The mail neuropsychological implications of social robotics in ASD treatment are:

1. Social cognition enhancement: One of the core challenges for individuals with ASD is in the domain of social cognition, which encompasses the ability to interpret, understand, and respond to social cues. Social robots, with their predictable and structured interactions, can provide a controlled environment for these individuals to practice and enhance their social cognitive skills (Pennisi et al., 2016).
2. Emotion recognition: Many individuals with ASD have difficulty recognizing and interpreting emotional expressions. Through facial recognition technology and programmed emotional expressions, robots like PEPPER can help these individuals practice emotion recognition in a consistent manner, bridging the gap between digital expressions and real-life human interactions (Cabibihan et al., 2013).
3. Theory of mind and perspective taking: Theory of Mind refers to the ability to attribute mental states to oneself and others. Through interactive scenarios, the robot can be programmed to exhibit certain “emotions” or “intentions”, allowing the individual with ASD to practice perspective-taking and understand that others may have thoughts and feelings different from their own (Scassellati et al., 2012).
4. Reducing anxiety: The predictability and consistency of robots can be less intimidating for some individuals with ASD compared to human interactions, which are often fraught with unpredictability. This can reduce anxiety and increase the willingness to engage in social interactions (Diehl et al., 2012).
5. Neuroplasticity and learning: Early and consistent interventions can harness the brain's neuroplasticity, especially in young individuals with ASD. By providing repetitive and structured social interactions, robots can potentially facilitate neural pathways associated with social cognition, enhancing the brain's capacity for social interactions (Wainer et al., 2014a).
6. Generalization to human interactions: While robots provide a controlled environment, the ultimate goal is to transfer the skills learned during robot-human interactions to human-human interactions. The structured learning environment can act as a bridge, helping individuals with ASD generalize their newly acquired skills to real-world scenarios (Tapus et al., 2012).
7. Ethical neuropsychological considerations: While the potential benefits are significant, it's crucial to ensure that the individual's cognitive and emotional wellbeing is prioritized. Over-reliance on robot interactions, for instance, might lead to reduced human-human interactions, which could be counterproductive in the long run.

In conclusion, the integration of social robotics into neuropsychological interventions for ASD offers a promising avenue to address core social and cognitive challenges faced by these individuals. However, it's imperative that such interventions are designed and implemented with a deep understanding of the neuropsychological underpinnings of ASD and are continuously monitored and adapted based on individual progress and wellbeing.

## 7. Limitations of the work and future implementations

Limitations of the work:

1. Generalizability: The scenarios presented are fictional examples to illustrate the potential of Pepper robot interactions for individuals with Autism Spectrum Disorder (ASD). The effectiveness and applicability of these scenarios may vary across individuals with ASD due to the wide spectrum of abilities and preferences within the disorder.
2. Individual variability: ASD is a complex and heterogeneous condition, and individuals with ASD may have unique needs and challenges. The presented scenarios may not address the specific requirements of every individual with ASD, necessitating customization and adaptation to suit individual differences.
3. Emotional recognition: While the integration of OpenAI's technologies and visual cues in the interactions assists individuals with impaired emotion recognition, it is important to note that it may not fully compensate for the challenges individuals with ASD may experience in perceiving and understanding emotions.

### 7.1. Technical limitations

The technologies and software used for the present work are frontier and still have technical limitations mainly attributable to rather long latency times. Specifically, the integration system of Pepper with OpenAI technologies, such as speech recognition, natural language understanding, and image generation, relies on the capabilities and limitations of the respective systems.

Against advantages such as flexibility in the responses generated and the ability to interact via Pepper's tablet there are disadvantages due to waiting, latency times also become longer when these technologies are combined in the different lessons presented. The main delays can be traced precisely to queries made to OpenAI's systems, specifically:

- The average chatGPT API response time is 15,000 ms;
- The response time of the speech recognition model, on the other hand, is closely related to the specific model size employed in the algorithm. in the case study discussed, the small size was employed, which has a transcription time of 16x the listening time.

These technologies may have inherent limitations in accuracy, processing speed, or availability of resources, which could impact the overall performance and effectiveness of the interactions. The benefits and flexibility of the system are such that these limitations are justified.

### 7.2. Future implementations

Future developments for this work are listed in this sub-section:

1. Personalization and adaptation: Future implementations could focus on developing personalized and adaptive interaction frameworks that consider the specific needs, preferences, and goals of individuals with ASD. Customization could involve tailoring the content, pacing, and level of support provided by the robot to suit each individual's unique requirements.
2. Enhanced Emotional understanding: Advancements in emotion recognition technologies could be incorporated into the interaction framework, enabling the robot to better perceive, interpret, and respond to the emotional cues of individuals with ASD. This could foster improved emotional understanding and empathy within the interactions.
3. Multimodal communication: Future implementations could explore the integration of additional communication modalities, such as gesture recognition or haptic feedback, to enhance the interaction experience for individuals with ASD. Incorporating these modalities could facilitate alternative forms of expression and engagement.
4. Long-term engagement and progress monitoring: Implementations could incorporate mechanisms to track and monitor the progress of individuals with ASD over time. Long-term engagement could allow for personalized interventions and ongoing assessment of communication skills, social interactions, and problem-solving abilities.
5. Collaboration with therapists and educators: Collaboration with professionals in the fields of therapy and education could provide valuable insights and guidance in the development and refinement of the interaction frameworks. This collaborative approach could help ensure that the implementations align with evidence-based practices and meet the specific goals of therapy or educational programs.

In conclusion, future implementations can focus on personalization, addressing individual variability, improving emotional understanding, incorporating multimodal communication, monitoring progress, and collaborating with professionals to enhance the effectiveness and applicability of Pepper robot interactions for individuals with ASD. These advancements have the potential to create more tailored, engaging, and supportive experiences for individuals with ASD, promoting their communication, social skills development, and problem-solving abilities.

## Data availability statement

The datasets presented in this study can be found in online repositories. The names of the repository/repositories and accession number(s) can be found below: https://drive.google.com/drive/folders/1q4GRwwRnqMwFKs0tLk2ZMBExhoerHp4c?usp=sharing.

## Author contributions

FB, PP, and EB developed the initial concept and theoretical framework. FD and FB were in charge of data collection and preliminary analysis. FB, FD, and CS provided detailed analysis and interpretation of data. EB and PP supervised the research process and ensured the quality of the work. All authors significantly contributed to this work. All authors were actively involved in the manuscript preparation and final approval, guaranteeing the accuracy, and integrity of the work.

## Supplementary material

The python code, for both versions employed 2.7 and 3.11, developed for the interactions described in this paper is available in the “PEPPER LESSONS” repository at the following link: https://drive.google.com/drive/folders/1q4GRwwRnqMwFKs0tLk2ZMBExhoerHp4c?usp=sharing.
