# Supplementary material for: A social robot connected with chatGPT to improve cognitive functioning in ASD subjects
Source: Front Psychol. 2023 Oct 5;14:1232177. doi: 10.3389/fpsyg.2023.1232177 (PMC10585023; doi:10.3389/fpsyg.2023.1232177)
Supplement: Supplementary file 1 [file Data_Sheet_1.pdf]

1 APPENDIX

Unit Title: ”Exploring the World with Pepper: An Adventure for Individuals with Medium-Functioning Autism Spectrum Disorder (ASD)” Objective of the Unit: The objective of this unit is to provide a personalized learning experience using the Pepper robot for individuals with medium-functioning Autism Spectrum Disorder (ASD). Through interactive activities and adapted materials, the unit aims to improve social skills, cognitive abilities, and interaction with the surrounding environment.

Estimated Duration: 4-6 weeks (may vary depending on individual needs)

Lesson 1: Introduction to Pepper and Building a Relationship

- Introduce Pepper as a friend and learning companion.
- Discuss the features of Pepper that might be interesting to the individual with ASD.
- Interactive activity: Facial recognition game with Pepper to help the individual become familiar with facial expressions and emotions.

Lesson 2: Exploring Emotions

- Discuss different emotions and recognizing corresponding facial expressions.
- Interactive activity: Emotion game with Pepper, where the individual can choose an emotion to express, and Pepper will respond accordingly.

Lesson 3: Basic Social Skills

- Introduction to basic social skills such as eye contact, active listening, and respecting personal space.
- Interactive activity: Simulate social situations with Pepper, where the individual can practice the learned skills through role-playing scenarios.

Lesson 4: Exploring the Surrounding World

- Utilize Pepper to explore and interact with the surrounding environment.
- Interactive activity: Treasure hunt with Pepper, where the individual can follow Pepper’s instructions to find specific

Lesson 5: Enhancing Cognitive Skills

- Problem-solving activities and personalized cognitive stimulation.
- Interactive activity: Puzzle games with Pepper to develop cognitive skills such as logical reasoning and memory.

Lesson 6: Review and Reflection

- Review acquired skills and abilities throughout the entire learning journey.
- Interactive activity: Q& A session with Pepper to allow the individual to ask questions and reflect on the learning experience with the robot.

Note: Throughout the entire unit, it is essential to pay attention to the individual preferences of the individual with ASD and personalize the activities accordingly. The support of teachers or therapists specialized in ASD can be crucial in adapting the activities.

Practical Examples:

1. During the facial recognition game, Pepper can display various expressions like happiness, sadness, or surprise, and the individual can try to identify and match the corresponding emotion.
2. In the social skills activity, Pepper can simulate a conversation, and the individual can practice making eye contact, actively listening, and responding appropriately to Pepper's prompts.
3. For the treasure hunt activity, Pepper can provide verbal instructions and guide the individual to search for specific objects in their immediate surroundings, such as finding a red ball or a blue book.
4. During the puzzle games, Pepper can present the individual with visual puzzles or riddles to solve, adjusting the difficulty level based on their cognitive abilities.
5. In the reflection session, the individual can engage in a conversation with Pepper, sharing their thoughts, experiences, and what they have learned throughout the unit. Pepper can provide encouragement and ask questions to prompt further reflection.
